# Relationships between ICT competencies related to work, self-esteem, and self-regulated learning with engineering competencies

**DOI:** 10.1371/journal.pone.0260659

**Published:** 2021-12-02

**Authors:** Buratin Khampirat

**Affiliations:** Institute of Social Technology, Suranaree University of Technology, Nakhon Ratchasima, Thailand; Rzeszow University of Technology: Politechnika Rzeszowska im Ignacego Lukasiewicza, POLAND

## Abstract

The rapid development of advanced technology worldwide has promoted an increase in the need for highly skilled engineers who are adept at applying job-related technologies and have engineering competency (ENcom) to gain knowledge and introduce creative solutions. However, little is known about the underlying mechanism of the associations between ICT competencies related to work (ICT-Work) and the ENcom of engineering students. This study sought to examine the role of ICT-Work on ENcom. Based on the literature, self-esteem and self-regulated learning (SRL) were identified as factors that indicate the effect of ICT-Work on ENcom, while gender was identified as a moderator that conditioned these mediated relationships. The sample consisted of 1,313 undergraduate engineering students from eleven universities in Thailand. The results of structural equation modeling (SEM) showed positive direct and indirect effects of ICT-Work on ENcom, self-esteem, and SRL and confirmed that self-esteem and SRL mediate the impact of ICT-Work on ENcom. Moreover, multigroup SEM revealed no gender differences in the factor loadings and structural path coefficients of ICT-Work on ENcom via self-esteem and SRL. To prepare students for their professional lives in the digital world, educational institutions should emphasize the importance of developing engineering students in ICT-Work and the use of advanced ICT involved in the job.

## Introduction

The digital society and the COVID-19 pandemic have changed the way people live, work, and learn around the world [1–3]. The workforce has an increasing demand for ICT competencies as a tool for knowledge-seeking, communication, learning, working, and shaping career choices [4, 5]. In some jobs, basic ICT skills are not specific to the profession; however, many job profiles in engineering industries increasingly require engineers with good technical expertise [6] and high-level ICT-Work and cross-functional skill capabilities [7] to apply specific software in their profession [8]. Merely possessing basic ICT skills is not sufficient because the labor market in the digital age and in the critical COVID-19 situation requires more technology capabilities and high-skill competencies [9]. Therefore, engineering students need to have sufficient knowledge and skills in using complex ICT systems for their profession in the labor market and for career development.

ICT competencies are essential to students in any career field, especially for engineering students, whose subject matter is difficult to study. Studies have reported that ICT competencies in curricula would benefit learning in terms of both cognitive and noncognitive development [10, 11], for example, increased higher-order thinking capacities [9, 12], self-efficacy [13], learning processes [14], academic performance [13, 15], and facilitating the development of students’ work skills [16]. In contrast, a study by Meng, et al. [17] found a significantly negative association between perceived ICT competencies and student achievement in China. Therefore, there is a gap in previous studies regarding the relationship between ICT competencies and academic performance.

According to Yardi and Bruckman [18], ICT-Work is important to prepare and motivate students for future work performance. Numerous studies have found that engineering graduates are unable to meet the requirements of the corporate world (e.g., OECD [4]; Saad and Majid [19]; Winberg, et al. [20]), as they lack ICT skills and competencies at an advanced level [21–23].

In Thailand, universities have made various efforts to promote the development and application of advanced ICTs that are appropriate for specific jobs; however, Thai students continue to lack ICT skills. This problem is not confined to Thailand only, and numerous studies in the Asia Pacific region and other parts of the world have shown that most graduates still have ICT competencies that are not well aligned with the jobs they are seeking [24–26]. Furthermore, Sa-Nguanmanasak and Khampirat [27] reported that Thai graduates lacked the development of ICT-Work competencies to keep pace with fast-changing digital work.

Despite growing interest in the human skills related to ICT, the processes by which ICT competencies affect human resource development in psychological aspects and career readiness of university students are limited, especially for engineering students who are faced with the challenges of innovation in modern industries and technology [28].

Several studies exist on the relationship of ICT competencies and self-esteem, self-regulated learning (SRL) [29], and learning performance; however, their relevance in explaining ICT-Work and these variables in the context of engineering education is limited. There is still a need to understand the impact of the different competencies of ICT on developing the potential of learners.

Taking into account the current context of the need for ICT-Work and the relevance of students’ ICT-Work perceptions about their ENcom, previous research has shown that little work has been undertaken to study the role of ICT-Work, which is a key factor that can affect job performance and career choices. Therefore, it is necessary to develop and study the impact of ICT-Work on ENcom. Such results may guide policymakers to make sound decisions about developing students with professional ICT skills to prepare them to work in the digital world.

Considering the above situation, the purposes of this study were (1) to propose and validate a framework of ICT-Work to predict ENcom, which mediates the effects of self-esteem, and SRL. The purpose of this prediction was to obtain a better understanding of the degree of ICT-Work knowledge and skills relevant to the professional development of engineering students and (2) to verify whether students’ gender was different in their perceptions of the impact of ICT-Work on ENcom.

This research primarily aims to address the following research questions:

RQ1: What are the explanatory and predictive patterns among students’ ICT-Work, self-esteem, SRL, and ENcom?
RQ2: Is there any difference between male and female students in terms of the relationships among these factors?

## Hypothesis development

### Self-determination theory

Self-determination theory (SDT) [30] was used to support the relationships between the constructs in the theoretical framework. SDT emphasizes that autonomy, competence, and relatedness are the basic psychological needs of students [31]. A greater level of these three driving forces enables a person’s automatic motivations and determines attitude and behavior [32].

Several studies and meta-analyses have supported the relationships between SDT-based constructs that are related to student motivation and performance [33]. Gupta Kriti [34] used SDT as the framework to investigate the factors underlying the adoption of massive open online courses (MOOCs). Markwell, et al. [35] pointed out that constructs related to the SDT concept impact student placement, learning, and experiences. Zheng, et al. [36] showed that students’ basic psychological needs for autonomy, competence, and relatedness motivated them to engage actively in learning and to have better performance and academic achievements. Therefore, SDT is a reasonable framework with which to investigate the relationships of ICT-Work, self-esteem, SRL, and ENcom.

### Relationship between ICT-Work and ENcom

ICT-Work conceptualizes knowledge, skills, and abilities domains that are needed for students to implement ICT in working processes to perform successfully in their field. There are several ways to enhance students’ competence and employability, where basic ICT skills and professional-related ICT may be one way [37]. Studies have argued that ICT competencies allow students to acquire new knowledge and to better adapt to the learning environment. Ni and Chen [38] proposed ICT competence for training students to succeed in their profession as multidimensional, encompassing knowledge, skills, and personal attributes that enable a person to achieve effectiveness at the individual, organizational, and professional levels. Using technology-based learning and building technology capacity enhances lifelong learning skills and improves competence in a specific field [39]; it also minimizes the gap between knowledge-oriented education and labor market needs [40]. Pirzada [41] emphasized that ICT-Work is associated with employability, and it could increase productivity in organizations and create better citizens. This can affect economic growth according to Bilan’s research [42], which demonstrated that long-term socioeconomic progress is related to characteristics of continuous digital development. Especially, for developing countries, developing a better ICT can help boost economic progress and financial efficiency. The ICT development should focus on increasing the accessibility of the Internet and efficient use of online technologies for individuals, households, and businesses [42].

When considering indirect relationships, previous studies demonstrated that university students with high ICT-Work capabilities exude higher self-esteem and have greater professional competence [43, 44]. Likewise, Shopova [45] reported that the development of the ICT competencies of university students is crucial for improving the effectiveness and efficiency of the learning process as well as for improving students’ ability to work in the dynamically changing labor market. However, the relationship between ICT skills and performance is not clear. Recently, the results of multilevel SEM by Wu [46] revealed a significant negative indirect relationship between ICT skills and learning performance via attention problems and SRL.

### Relationship between ICT-Work and SRL

The development of students’ SRL could be facilitated by ICT usage and ICT literacy [47]. Students who are able to use ICT for tasks are more likely to persist in effective learning strategies and have a greater chance of achieving successful academic results than those that do not use ICT [48, 49]. Likewise, longitudinal mixed methods of Muthupoltotage and Gardner [50] concluded that students’ digital literacy affects some SRL and that they are reciprocal relationships. Zylka, et al. [51] reported that there was a positive correlation between ICT engagement and metacognitive processes. Some studies have shown that SRL is effective for ICT skills [52, 53]. Greene, et al. [54] and Demirbag and Bahcivan [55] noted that SRL plays a vital role in developing learners’ digital skills. Due to the relationship between SRL and ICT-Work, it is not clear what factor is causing the effect. Therefore, it is important to investigate how ICT-Work can support SRL among engineering students to confirm the above findings.

### Relationship between ICT-Work and self-esteem

The concept of self-esteem refers to an individual’s judgment of one’s self-worth and is associated with learning outcomes [56]. Hale, et al. [57] and Youssef and Dahmani [44] stated that ICT-Work can promote a deeper understanding of facts and increase self-esteem, because it affects a person’s way of thinking about themselves based on their abilities. Most of the employers and students agreed that the knowledge and skills in ICT-Work would increase their employability [58]. For example, ICT gives learners the opportunity to access learning resources and work skills that increase their sense of self-esteem [57]. Contemporary workplaces need digital-savvy potential employees who can work efficiently and smoothly through constantly updating ICT [59] with a high level of self-esteem to increase performance.

### Role of SRL on self-esteem

Following SDT [30], intrinsic motivation and self-regulation for expressing a specific behavior were positively correlated with self-esteem [60]. When taking action, students regulate their behavior to achieve their desired goals [61]. Studies have shown that students with high self-regulation are identified as having direct ties to self-esteem and life satisfaction [62, 63].

### Role of SRL on ENcom

SRL is defined as a learning method that is guided by metacognition, strategic execution, and learning motivation [64, 65], which are strategies a learner would like to practice in studying to enhance academic success [66, 67]. Students with more SRL become more effective learners who are self-motivated and achieve more [68]. Phan [69] and Platow, et al. [70] found that students’ self-regulated deep learning was positively related to academic performance. Nelson, et al. [71]; Zheng, et al. [72] also revealed the importance of SRL to student performance in engineering learning. However, Zheng, et al. [73] suggested that mobile SRL enhanced students’ learning achievements and SRL skills. Since SRL is an important predictor of academic performance [74], it is important to expand the findings on how SRL affects ENcom.

### Relationship between self-esteem and ENcom

Job demands-resources (JD-R) theory [75, 76] hypothesizes that personal resources (i.e., self-esteem, hope, resilience, proactive personality, high levels of energy) are a crucial factor that affects self-regulation, work engagement, satisfaction, well-being, and job performance. Self-esteem plays an important role in student development [56]. Empirical studies in students have shown that self-esteem is positively correlated with competence [77, 78] and academic performance [79–81]. Additionally, a study by Barros and Duarte [82] revealed that self-worth has a positive effect on academic achievement. Johnson, et al. [83] agreed that high self-esteem is associated with high career aspirations and intellectual and academic competence that are linked to their professional values. Fényes et al. [84] investigated students’ persistence in higher education and their motivation for further study in five countries of Central and Eastern Europe, revealing that career consciousness of students is positively related to commitment to graduation and further studies. Fényes et al.’s study also showed that male students are less concerned about their careers and less persistent than females. Self-esteem tends to have a positive influence on performance and job engagement because individuals with high self-esteem view themselves as competent and productive and are more likely to seek challenges contributing to personal growth in work activities [85, 86].

### Moderating effects of gender

In studying the variation in the degree of relationships between constructs, gender differences are the most reported factors among university students [87]. Sobieraj and Krämer [88] investigated gender differences in competence and study motivation regarding STEM subjects, reporting that males perceived more self-efficacy and leadership aspirations than females. In a longitudinal study of STEM undergraduates, MacPhee, et al. [89] reported that at the time of admission, males scored themselves higher on academic skills than females. By the time of graduation, males’ academic self-concept was equal to that of females. However, Nagahi, et al. [90] found no gender differences in the relationship between engineering students’ systems thinking skills, five-factor model personality traits, proactive personality scale, and academic performance. Pop and Khampirat [91] also revealed no difference between male and female graduates in employability, except for achievement motives, where males rated themselves higher than females. Motahhari-Nejad [92] tested the measurement invariance of professional competencies in engineering students across genders, indicating that there was consistency across genders in the model. Referring to previous findings, differences between males and females in their behavior and competencies should be further understood, and it is important to examine how the relationships among ICT-Work, self-esteem, SRL, and ENcom would be similar or differ by gender.

Based on these previous studies, the following research hypotheses are formulated:

**Hypotheses on direct effects:**

**H1:** ICT-Work has a positive impact on ENcom.
**H2:** ICT-Work has a positive impact on SRL.
**H3:** ICT-Work (H3a) and SRL (H3b) have a positive impact on self-esteem.
**H4:** SRL (H4a) and self-esteem (H4b) have a positive impact on ENcom.

**Hypotheses on indirect effects:**

**H5.** There are mediated relationships between ICT-Work and ENcom through SRL and self-esteem.
**H6.** There are mediated relationships between SRL and ENcom through self-esteem.

**Hypothesis on the multigroup model:**

**H7:** The relationship between ICT-Work and ENcom via self-esteem and SRL was not different between male and female student groups.

**Fig 1:**
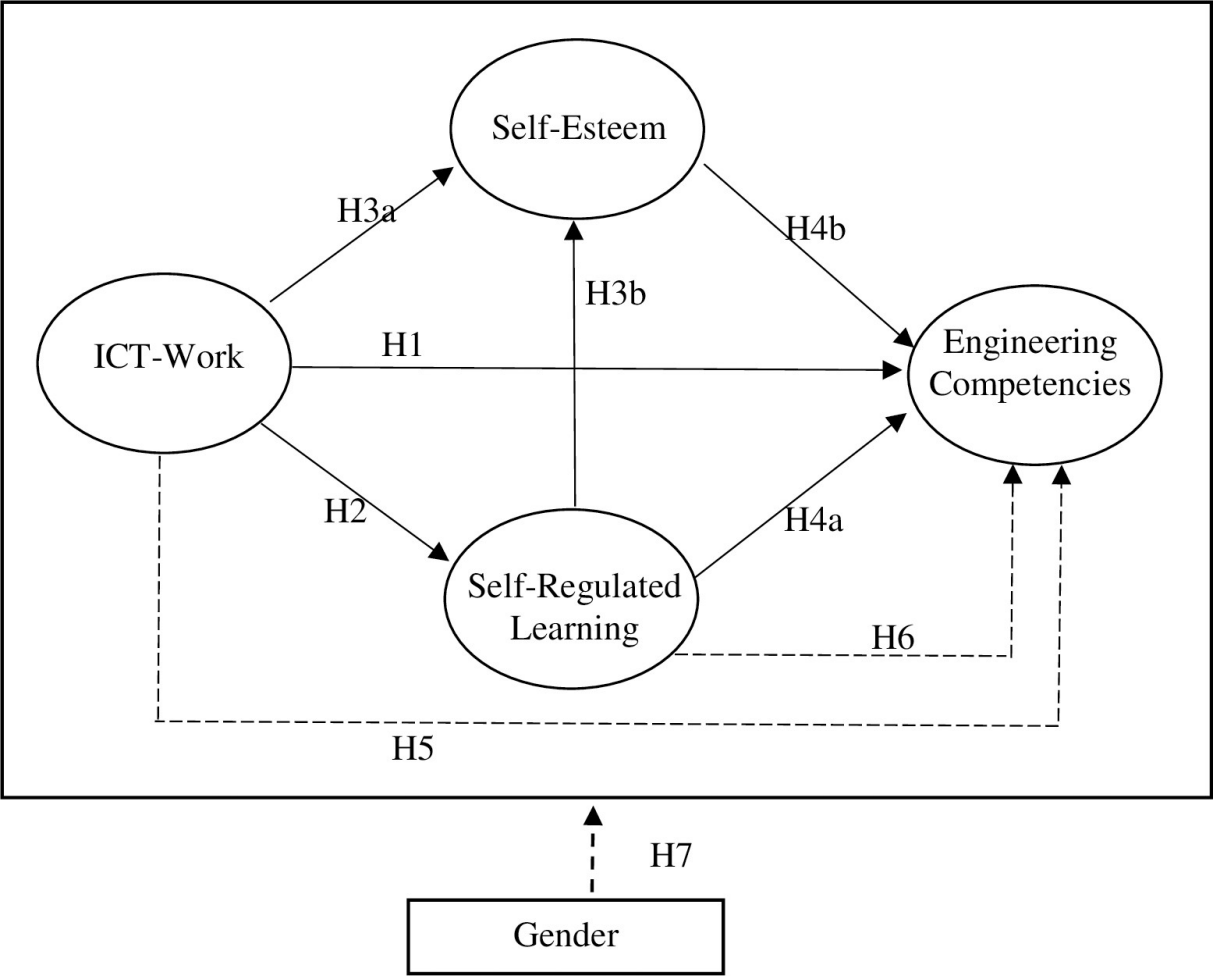
Proposed theoretical model of the effects of ICT-Work on ENcom. (In which there were the moderating effects of gender. The long dashed lines indicate indirect effects and multigroup analysis.).

Therefore, a research framework composed of the above hypotheses is constructed, as illustrated in Fig 1.

## Materials and methods

### Participants

The sample consisted of 1,313 (508 females and 805 males) engineering undergraduate students from eleven universities in Thailand. Their mean GPA was 2.72 (*SD* = 0.43) for females and 2.69 (*SD* = 0.47) for males. Students’ average age was 21.98 years old (Min. = 18, Max. = 34, and *SD* = 1.29 years old), and the mean workplace internship experience was 3.92 months. Approximately 65% of the participants whose family average monthly income was ≤ 60,000 Baht (1,666.67 USD) (Table 1).

**Table 1:** Demographic characteristics of the participants by gender (N = 1,313).

Variable	Categories	n	%
Gender	Female	508	38.69
	Male	805	61.31
GPA (scale ranged from 0 to 4.0) (*M* = 2.71, *SD* = 0.45)	< 2.00	22	1.68
	2.00–2.50	465	35.42
	2.51–3.00	463	35.26
	3.01–3.50	263	20.03
	> 3.50	68	5.18
	Missing	32	2.44
Family Income (per month) (Note: 32.00 THB = 1 USD)	≤ 10,000 THB (≤ 333.33 USD)	147	11.20
	10,001–20,000 THB (333.37–666.67 USD)	263	20.03
	20,001–30,000 THB (666.70–1,000.00 USD)	215	16.37
	30,0001–40,000 THB (1,000.03–1,333.33 USD)	108	8.23
	40,001–50,000 THB (1,333.37–1,666.67 USD)	120	9.14
	50,001–60,000 THB (1,666.70–2,000.00 USD)	55	4.19
	60,001–80,000 THB (2,000.03–2,666.67 USD)	72	5.48
	80,001–100,000 THB (2,666.70–3,333.33 USD)	81	6.17
	100,001–200,000 THB (3,333.37–6,666.67 USD)	47	3.58
	> 200,001 THB (> 6,666.70 USD)	15	1.14
	Missing	190	14.47

### Instruments

#### ICT competencies related to work (ICT-Work)

The engineering student assessment for ICT-Work consisted of 8 items that assess the extent to which the students consider themselves to have the knowledge and skills in applying advanced computers and ICT and professional tools in engineering practice to different work situations. For example, “can interact with cutting-edge software interfaces such as human-machine interfaces, human-robot interaction, etc.”. The items were measured on a 5-point Likert scale (1 = strongly disagree, and 5 = strongly agree).

#### Self-esteem

Students’ self-esteem was measured by using the questionnaire developed by Rosenberg [93]. The Rosenberg self-esteem scale consists of 10 items developed to assess positive and negative evaluations of feelings about the self as a one-dimensional construct, such as “I feel that I have a number of good qualities”. Participants responded on a 4-point Likert scale ranging from 1 (strongly disagree) to 4 (strongly agree).

#### Self-regulated learning (SRL)

The questionnaire of Pintrich, et al. [94] was adapted to assess students’ SRL in this study. The scale included 5 items, such as “I work hard to do well in this class even if I do not like what we are doing”. Students rated all the items on a 5-point Likert scale ranging from 1 (not at all true) to 5 (strongly agree).

#### Engineering competencies (ENcom)

The ENcom scale was designed and developed by the author based on previous studies, the framework of ABET, and engineering students’ learning outcomes. The scale comprises 13 items (e.g., “can apply engineering process, technics, and design to solve the engineering problem effectively”). All items were rated on a 5-point Likert-type scale, ranging from 1 (strongly disagree) to 5 (strongly agree).

The internal consistency reliability (Cronbach’s α) in the total sample and classified by gender ranged from 0.84 to 0.85 for ICT-Work, 0.73 to 0.76 for self-esteem, and 0.87 to 0.89 for ENcom, which met the benchmark level [95], except for SRL (0.52 to 0.58), which were smaller than the acceptable values (Table 3). However, the author still continued to use the SRL scale because the value for Cronbach’s alpha is affected by the number of items in the scale [96]. The questionnaires in English and Thai were included as S1 Questionnaire.

### Procedure

Before collecting data, permission for the student to participate in the survey was granted by universities and decision-makers. This study was approved by the Ethics Committee of Suranaree University of Technology, Thailand (EC-61-93). Verbal informed consent was obtained from all participants in this study. Participants had given their consent and were informed that their information would only be used for research purposes and that their participation would not affect grades in the course. The information provided was voluntary and anonymous. If the participants were uncomfortable completing the questionnaire, they could terminate independently.

### Data analysis

Preliminary analyses were conducted to screen the data for univariate and multivariate normality, homoscedasticity, and multicollinearity using SPSS 18.0. For sample sizes greater than 300, values of absolute skewness < 2 and absolute kurtosis < 7 were considered indicative of univariate normality [97, 98]. Whereas multivariate skewness and kurtosis were assessed by Mardia’s multivariate analysis of skewness and kurtosis, if *p*-values for Mardia’s coefficients were greater than 0.05, then multivariate normality was accepted [99, 100]. The Kaiser-Meyer-Olkin Measure of Sampling Adequacy (KMO, should be ≥ 0.5) was applied as an index for investigating whether data and sample size were sufficient for performing factor analysis [101]. Bartlett’s test of sphericity was used to verify the homoscedasticity or the constant variance of error terms across samples and whether the correlation matrix is an identity matrix (in other words, it is a redundancy between variables). A *p*-value less than 0.05 indicates that variables are unrelated and therefore unsuitable for factor analysis [102]. Tolerance and the variance inflation factor (VIF) were used to detect multicollinearity in a set of variables in the model [103]. Tolerance values < 0.10 or VIF values > 10 indicated multicollinearity problems [104].

Descriptive statistics were calculated to summarize aspects of participants and variables, whereas Pearson correlation coefficients were measured to determine the strength and direction of a linear relationship between two variables.

Confirmatory factor analysis (CFA) was conducted to evaluate the construct validity of the measurement model [105]. Structural equation modeling (SEM) was then employed to investigate the direct and indirect relationships between constructs in the theoretical model and test measurement invariance in gender subgroups [106]. CFA and SEM were performed using Mplus 8.3 [107]. The model fits to the data were assessed using the following goodness-of-fit indices: relative chi-square (*χ2/df*, acceptable if < 3) [108], root mean square error of approximation (RMSEA, acceptable if < 0.08) [109], standardized root mean square residual (SRMR, acceptable if < 0.08), comparative fit index (CFI, acceptable if ≥ 0.90), and Tucker-Lewis index (TLI, acceptable if ≥ 0.90) [108, 110].

To test the multigroup measurements and structural models, the SEM of the total sample and of each group is tested first when the baseline model of each group is fit to the data. The next step is to determine the equivalence of the model form or configural invariance. If configural invariance is supported, then the measurement and structural models are comparable across the groups using a hierarchical sequence of nested models [111]. The change in the *χ*^2^ value (Δ*χ*^2^) was used to evaluate the invariance between the two nested models. However, because Δ*χ*^2^ is sensitive to sample size [112, 113], the cutoff points of the very small difference in CFI (ΔCFI ≤ .010), RMSEA (ΔRMSEA ≤ .015) or SRMR (ΔSRMR ≤ .030) [113, 114] between two models were considered to indicate that in a large sample (>300), the invariance hypothesis should not be rejected [112]. In addition, this study also used Akaike information criterion (AIC) and Bayesian information criterion (BIC) values to evaluate the multigroup variance according to the suggestion of Schoot, et al. [115] and Vrieze [116]. Lower AIC or BIC values indicate that a better fit, complexity, and invariance can be supported [115].

## Results

### Descriptive statistics and preliminary analyses

The dataset used in the analysis of the relationship between ICT competencies related to work, self-esteem, and self-regulated learning with engineering competencies was included as S1 Dataset. The minimum (Min.), maximum (Max.), mean (*M*), standard deviation (*SD*), skewness (*SK*), and kurtosis (*KU*) for all variables and measures are given in Table 2. The mean score of ICT-Work was 3.61 (*SD* = 0.51), self-esteem was 2.96 (*SD* = 0.39), SRL was 3.23 (*SD* = 0.53), and ENcom was 3.72 (*SD* = 0.44). SK ranged from −0.07 to 0.65, and KU ranged from −0.54 to −0.35; absolute skew values were less than 2 (*SK* < 2) and absolute kurtosis values were less than 7 (*KU* < 7), and p-values for Mardia’s coefficients > 0.05 indicating that the data are multivariate normally distributed. The value of KMO (KMO = 0.780) and Bartlett’s test of sphericity (*χ2* = 2027.864, *df* = 66, *p* < 0.001) were within the recommended range, supporting the use of factor analysis [117].

**Table 2:** Bivariate correlations, descriptive statistics, and reliability indices of the latent measures for the whole sample (N = 1,313).

Latent Variables	No. of Items	Min.	Max.	*M*	*SD*	*SK*	*KU*	ICT-Work	Self-Esteem	SRL	ENcom
ICT-Work	8	2	5	3.61	0.51	0.17	-0.13		.145**	.167**	.708**
Self-Esteem	10	1.6	3.8	2.96	0.39	-0.07	-0.54	.186**		.356**	.322**
SRL	5	1.8	5	3.23	0.53	0.65	0.26	0.050	.401**		.303**
ENcom	13	1.92	5	3.72	0.44	0.01	0.35	.749**	.292**	.131**	

Note:

* *p* < .05

** *p* < .01; correlation coefficients above the diagonal are the correlations between constructs among female students; correlation coefficients below the diagonal are the correlations between constructs among male students.

The Pearson’s correlation coefficient (*r*) measures the linear relationship between the constructs are given in Table 2, and all measured variables were positively correlated. ICT-Work was more strongly and positively correlated with ENcom both in female (*r* = .708) and male students (*r* = .749), followed by self-esteem (*r* = .145 for female; *r* = .186 for male). However, in SRL, ICT-Work showed a significant correlation with SRL only in the female student group (*r* = .167). For relationships between other constructs in the model, such as self-esteem and SRL or SRL and ENcom, it was found that all pairs had a positive correlation (*p* < .01). Moreover, the values of tolerance and VIF were > 0.10 and < 10.00, respectively, indicating the absence of multicollinearity.

### Measurement model in the proposed model

**Fig 2:**
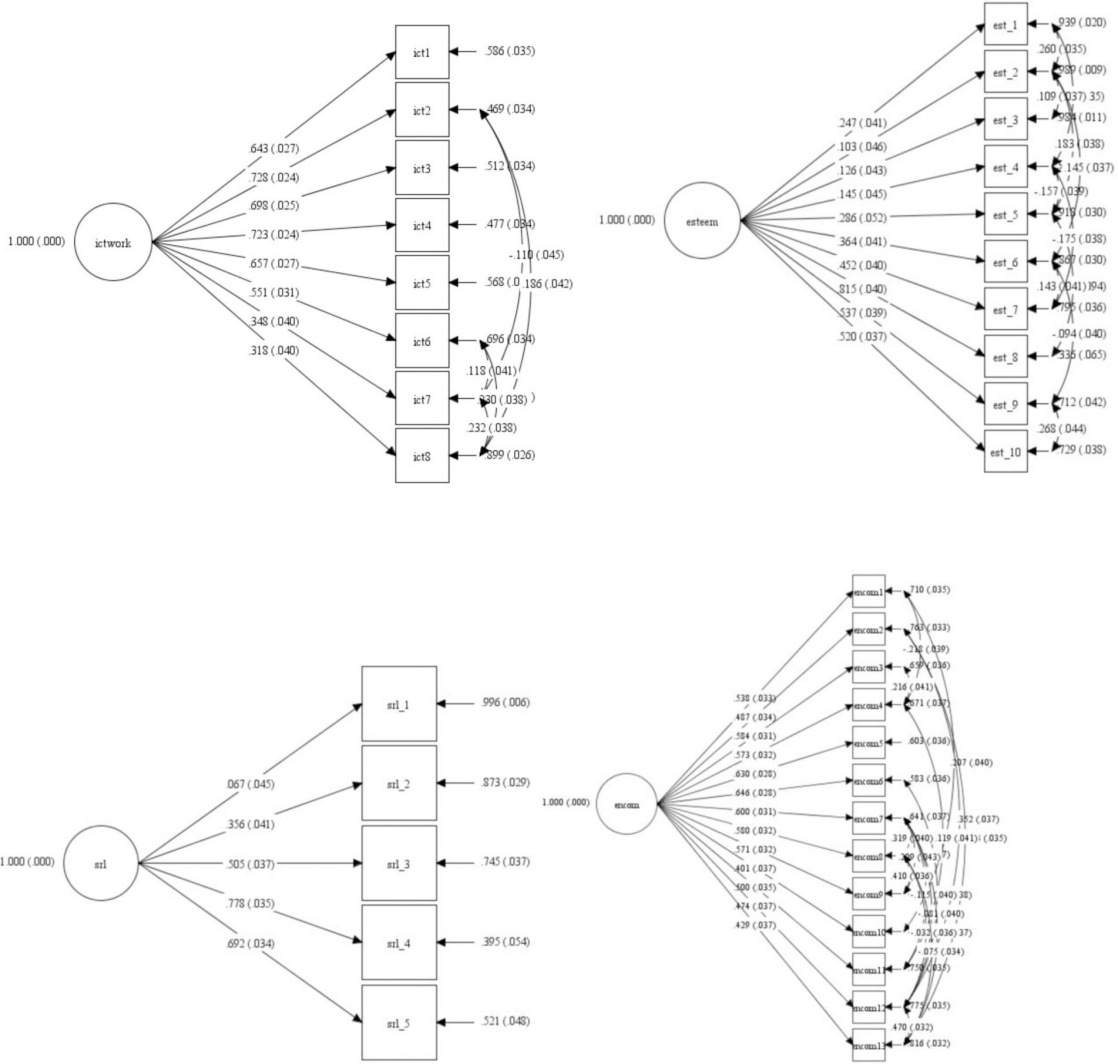
The standardized factor loadings of the common factor CFA for the total sample. Note: The circles and rectangles represent the latent factors and items, respectively; the single-headed arrows pointing from the latent factors to each item represent standardized factor loadings (standard errors are in parentheses).

CFA was performed to test the measurement properties of the four scales based on the total sample and gender. Goodness-of-fit indices for all measurement models, which appear in Table 3, showed good fits to the data for ICT-Work, self-esteem, SRL, and ENcom. These results indicated that the four scales were correlated with each of the established measures. The standardized factor loadings of each item are shown in Fig 2.

**Table 3:** Fit indices of the measurement factor models for the whole sample and student gender.

Measurement Model	Gender	Absolute fit indices						Incremental fit indices		Cronbach’s α	AVE	CR
		*χ* ^2^	*df*	*p*	*χ*^2^/*df*	RMSEA (90% CI)	SRMR	CFI	TLI			
ICT-Work	Total	40.67	15	0.00	2.71	0.05 (0.03–0.07)	0.02	0.98	0.97	0.85	0.36	0.81
	Male	41.62	15	0.00	2.77	0.07 (0.04–0.09)	0.03	0.97	0.95	0.85	0.38	0.82
	Female	23.88	12	0.02	1.99	0.04 (0.02–0.07)	0.03	0.99	0.98	0.84	0.36	0.80
Self-Esteem	Total	52.06	24	0.00	2.17	0.04 (0.03–0.06)	0.03	0.97	0.94	0.74	0.18	0.61
	Male	55.36	28	0.00	1.98	0.05 (0.03–0.07)	0.04	0.95	0.93	0.73	0.20	0.63
	Female	66.10	23	0.00	2.87	0.06 (0.04–0.08)	0.04	0.97	0.94	0.76	0.29	0.66
SRL	Total	15.04	5	0.01	3.01	0.05 (0.02–0.09)	0.03	0.98	0.95	0.55	0.29	0.62
	Male	14.54	5	0.01	2.90	0.07 (0.03–0.11)	0.03	0.97	0.94	0.52	0.31	0.64
	Female	9.14	4	0.06	2.29	0.05 (0.01–0.10)	0.03	0.98	0.95	0.54	0.25	0.52
ENcom	Total	118.02	49	0.00	2.41	0.05 (0.04–0.06)	0.03	0.97	0.96	0.88	0.30	0.84
	Male	73.46	49	0.01	1.50	0.04 (0.02–0.05)	0.03	0.99	0.98	0.89	0.32	0.86
	Female	112.69	48	0.00	2.35	0.05 (0.04–0.06)	0.04	0.97	0.95	0.87	0.32	0.85

Note: AVE = Average Variance Extracted, CR = Composite Reliability.

Regarding the convergent validity, composite reliability (CR) for each construct met the acceptable level of 0.6 [104], except for the female SRL. The average variance extracted (AVE) was between 0.18 and 0.38, which was lower than the standard 0.5 recommended by Hair, Black, Babin and Anderson [104] (see Table 3). However, when AVE is less than 0.5 but CR is greater than 0.6, the construct’s convergent validity is still sufficient, according to Fornell and Larcker [118] and Khampirat [119]. We should accept the AVE of these constructs because the CR exceeded the recommended standard.

### Mean differences by gender

The results of the independent t-test used to compare the mean scores for the four factors in the research framework are shown in Table 4. As the results show, there were statistically significant differences for the four factors as follows: female students perceived that they had higher scores than male students in ICT-Work (*M*_female_ = 3.68, *M*_male_ = 3.50; *t*(1144.74) = 6.50, *p* < 0.001, *d* = 0.36) and ENcom (*M*_female_ = 3.75, *M*_male_ = 3.67; *t*(1153.34) = 3.60, *p* < 0.001, *d* = 0.20). On the other hand, male students demonstrated higher scores than female students in self-esteem (*M*_female_ = 2.93, *M*_male_ = 3.00; *t*(1133.87) = 3.09, *p* < 0.01, *d* = 0.17) and SRL (*M*_female_ = 3.16, *M*_male_ = 3.32; *t*(1029.63) = 5.32, *p* < 0.001, *d* = 0.30).

**Table 4:** Comparison of ICT-Work, self-esteem, SRL, and ENcom scores by gender.

Latent Variables	Gender	*M*	*SD*	Mean difference	t-Test	*p*-value	Cohen’s d
ICT-Work	Male	3.50	0.47	0.18	-6.50***	0.000	0.36
	Female	3.68	0.51				
Self-Esteem	Male	3.00	0.37	0.07	3.09**	0.002	0.17
	Female	2.93	0.40				
SRL	Male	3.32	0.54	0.16	5.32***	0.000	0.30
	Female	3.16	0.51				
ENcom	Male	3.67	0.41	0.08	-3.60***	0.000	0.20
	Female	3.75	0.45				

Note.

** *p* < .01

*** *p* < .001.

### Direct and indirect effects of ICT-Work, self-esteem, and SRL on ENcom

**Fig 3:**
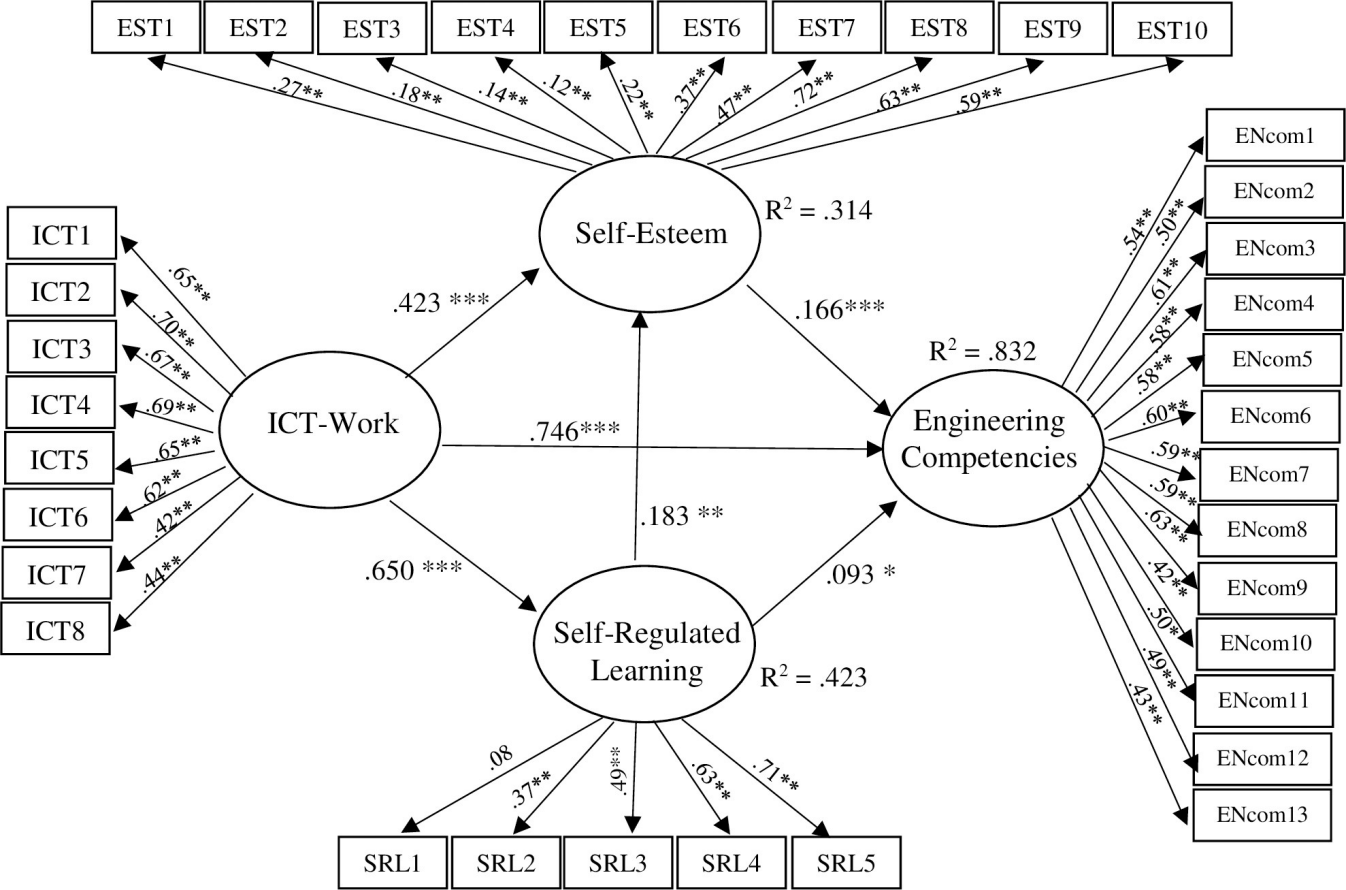
Structural equation model baseline for the whole sample. All coefficients are standardized. Note. * *p* < .05, ** *p* < .05, *** *p* < .001. The ellipses and rectangles represent the latent factors and items (or observed variables), respectively; the single-headed arrows pointing from latent factors to latent factors show regression paths or the impact of the predictors on the outcome variables; the single-headed arrows pointing from the latent factors to each item represent standardized factor loadings.

The SEM results for the total sample showed that the proposed model fit the data adequately, χ2 = 1622.07, *df* = 548, p < 0.001, χ2/*df* = 2.96, RMSEA = 0.06 (90% CI: 05 to 0.06), CFI = 0.86, TLI = 0.83, SRMR = 0.06 (Table 5 and Fig 3), and all the explanatory variables explained 83% (*R*^*2*^ = 0.83) of the variance in ENcom.

**Table 5:** Direct, indirect, and total paths of the conceptual framework (N = 1,313).

Predictor	Outcome	Mediator(s)	Direct		Indirect		Total Paths	
			Coef.	Std. Coef.	Coef.	Std. Coef.	Coef.	Std. Coef.
ICT-Work	ENcom	Self-Esteem, SRL	0.690***	0.746***	0.018*	0.020*	0.829**	0.897***
		Self-Esteem			0.065***	0.070**		
		SRL			0.056*	0.060*		
ICT-Work	SRL	-	0.770***	0.650***				
	Self-Esteem	-	0.472 ***	0.423***				
SRL	Self-Esteem	-	0.173**	0.183**				
	ENcom	Self-Esteem	0.072 *	0.093*	0.024*	0.030*	0.096*	0.123*
Self-Esteem	ENcom	-	0.138***	0.166***				

Note.

* *p* < .05

** *p* < .01

*** *p* < .001; Coef. = unstandardized path coefficients. Std. Coef. = standardized path coefficients.

Regarding the direct relationships, ICT-Work had a statistically significant positive effect on ENcom (H1: *β* = 0.76, *p* < 0.001), SRL (H2: *β* = 0.16, *p* < 0.05), and self-esteem (H3a: *β* = 0.45, *p* < 0.001). Thus, the H1, H2, and H3a were supported. This means that students who reported higher levels of perceived ICT-Work tended to report higher levels of ENcom, SRL, and self-esteem than their peers who reported lower levels of perceived ICT-Work. Similarly, SRL had a significant positive effect on self-esteem (H3b: *β* = 0.16, *p* < 0.05), and students who reported higher levels of SRL also tended to report higher levels of self-esteem. The SEM results also found that SRL (H4a: *β* = 0.76, *p* < 0.001) and self-esteem (H4b: *β* = 0.76, *p* < 0.001) also had statistically significant positive effects on ENcom. Students who reported higher levels of SRL and self-esteem also tended to report higher levels of ENcom.

Regarding the indirect effect, the analysis also supported both the indirect effect of ICT-Work on ENcom via SRL and self-esteem (H5: *β* = 0.020, *p* < 0.05) and the indirect effect of SRL on ENcom through self-esteem (H6: *β* = .030, *p* < .05). The SEM results for the total sample are summarized in Table 5, and standardized path coefficients are shown in Fig 3.

### Multigroup structural equation modeling analysis by gender

The SEM results obtained in the previous step may differ due to student gender. To investigate the similarities between female and male students of population parameters in the proposed model, multigroup analyses were performed, and gender was the grouping variable to assess whether the model form, factor loadings, and path coefficients in the hypothesized model were invariant across student gender.

The analysis started with testing the baseline model that was replicated separately for female and male students. The SEM for both male (χ2/*df* = 2.439, RMSEA = 0.042, CFI = 0.938, TLI = 0.921, SRMR = 0.075) and female (χ2/*df* = 2.100, RMSEA = 0.047, CFI = 0.908, TLI = 0.890, SRMR = 0.089) students yielded a sufficient fit to the empirical data (Table 6), and the same factor loadings were significant in the two groups.

**Table 6:** Model fit indices for multigroup analysis between male and female student groups.

Model	*χ* ^2^	*df*	*χ*^2^/*df*	RMSEA	SRMR	CFI	TLI	Comparison	Δ*χ*^2^	ΔCFI	ΔRMSEA	ΔSRMR	Decision
				(90% CI)									
**Baseline Model (no equality constraints imposed)**													
Whole sample	1370.219	537	2.552	0.049	0.050	0.888	0.868	-	-	-			Good Fit
				(0.045–0.052)									
Male sample	1202.481	493	2.439	0.042	0.075	0.938	0.921	-	-	-			Good Fit
				(0.039–0.045)									
Female sample	1111.020	529	2.100	0.047	0.089	0.908	0.890	-	-	-			Good Fit
				(0.043–0.050)									
**Multigroup Analysis**													
*Model form*													
MG1	1954.366	1090	1.793	0.049	0.061	0.885	0.867						Accepted
				(0.046–0.053)									
*Measurement model*													
MG2	2060.093	1134	1.817	0.050	0.063	0.877	0.863	1 vs 2	105.727	-0.008	-0.001	0.002	Accepted
				(0.046–0.053)									
*Structural model*													
MG3	2036.046	1139	1.788	0.049	0.065	0.869	0.855	2 vs 3	-24.047	-0.008	-0.001	0.002	Accepted
				(0.046–0.052)									
MG4	2076.878	1123	1.849	0.051	0.083	0.873	0.858	3 vs 4	40.832	0.004	0.002	0.018	Accepted
				(0.047–0.054)									
MG5	2197.536	1145	1.919	0.053	0.066	0.860	0.846	4 vs 5	120.658	-0.013	0.002	-0.017	Accepted
				(0.050–0.056)									
MG6	2138.557	1142	1.873	0.052	0.065	0.868	0.854	5 vs 6	-58.979	0.008	-0.001	-0.001	Accepted
				(0.048–0.055)									
MG7	2131.152	1123	1.898	0.052	0.065	0.866	0.850	6 vs 7	-7.405	-0.002	0.000	0.000	Accepted
				(0.049–0.056)									
MG8	2077.655	1129	1.840	0.051	0.075	0.874	0.859	7 vs 8	-42.298	0.008	-0.001	0.010	Accepted
				(0.047–0.054)									

Note. ***p < .001.; MG = multigroup.

MG1: All parameters of SEM free.

MG2: All factor loadings in the measurement model were constrained equal across groups.

MG3: The path coefficient from ICT-Work to self-esteem was constrained to be equal.

MG4: The path coefficient from ICT-Work to SRL was constrained to be equal.

MG5: The path coefficient from ICT-Work to ENcom was constrained to be equal.

MG6: The path coefficient from SRL to self-esteem was constrained to be equal.

MG7: The path coefficient from self-esteem to ENcom was constrained to be equal.

MG8: The path coefficient from SRL to ENcom was constrained to be equal.

Next, multigroup SEM was examined, starting from examining the invariance of the model form or configural invariance (MG1). In Table 6, the results showed that the model form fit each group separately (*χ*^2^/*df* = 1.793, RMSEA = 0.049, CFI = 0.885, TLI = 0.867, SRMR = 0.061), without any equality constraints, indicating that the configural invariance model was acceptable. This means that the basic organization of the factor structure is similar in the two groups [120]. Then, the next step of factor loading and structural invariances were tested.

As seen in Table 6, when all factor loadings in the model were constrained to be equivalent across gender, the results showed that the model provides factor loadings invariance (MG2) across student gender. The differences in the goodness of fit statistics (ΔCFI, ΔSRMR, and ΔRMSEA) were less than 0.01, and the *χ*^2^/*df* was 1.817, which was less than 3.00. These results indicated that the model reached matric invariance between males and females, which means that each factor loading is similar across gender groups. Therefore, the equality of the structural model between male and female engineering student groups could be examined.

Similarly, in the previous step, the multigroup analyses revealed that the structural path coefficients for “ICT-Work to self-esteem (MG3)”, “ICT-Work to SRL (MG4)”, “ICT-Work to ENcom (MG5)”, “SRL to self-esteem (MG6)”, “self-esteem to ENcom (MG7)”, and “SRL to ENcom (MG8)” showed no difference in tendency between male and female engineering students. The differences in the goodness of fit statistics (ΔCFI, ΔSRMR, and ΔRMSEA) were less than 0.01, and the *χ*^2^/*df* values were less than 3.00. Thus, hypothesis H7 was supported. According to this result, structural path coefficients between latent factors in each gender are the same. The final model of multigroup SEM with invariance of all factor loadings and structural path coefficients between male and female engineering students is depicted in Table 7.

**Table 7:** The unstandardized parameter estimates of the final model of multigroup SEM in which all factor loadings and structural path coefficients between male and female engineering student groups are invariant.

Measurement Models/ Constructs	Female				Male			
	*B*	*S*.*E*.	*P*-value	Intercepts	*B*	*S*.*E*.	*P-*value	Intercepts
**Factor loadings are the same across gender groups**								
*ENcom*								
ENcom1	0.86	0.08	0.00	3.56	0.86	0.08	0.00	3.56
ENcom2	0.83	0.08	0.00	3.81	0.83	0.08	0.00	3.81
ENcom3	0.91	0.07	0.00	3.56	0.91	0.07	0.00	3.56
ENcom4	0.94	0.08	0.00	3.62	0.94	0.08	0.00	3.62
ENcom5	0.88	0.08	0.00	3.63	0.88	0.08	0.00	3.63
ENcom6	0.99	0.08	0.00	3.50	0.99	0.08	0.00	3.50
ENcom7	1.00	0.00	999.00	3.45	1.00	0.00	999.00	3.45
ENcom8	0.97	0.07	0.00	3.47	0.97	0.07	0.00	3.47
ENcom9	0.99	0.07	0.00	3.51	0.99	0.07	0.00	3.51
ENcom10	0.71	0.07	0.00	3.76	0.71	0.07	0.00	3.76
ENcom11	0.83	0.08	0.00	3.73	0.83	0.08	0.00	3.73
ENcom12	0.75	0.08	0.00	3.83	0.75	0.08	0.00	3.83
ENcom13	0.71	0.08	0.00	3.84	0.71	0.08	0.00	3.84
*SRL*								
SRL_1	0.16	0.09	0.07	3.25	0.16	0.09	0.07	3.25
SRL_2	0.72	0.09	0.00	3.22	0.72	0.09	0.00	3.22
SRL_3	0.87	0.09	0.00	3.31	0.87	0.09	0.00	3.31
SRL_4	1.00	0.00	999.00	3.44	1.00	0.00	999.00	3.44
SRL_5	1.08	0.10	0.00	3.48	1.08	0.10	0.00	3.48
*Self-Esteem*								
EST_1	0.42	0.07	0.00	3.07	0.42	0.07	0.00	3.07
EST_2	0.28	0.07	0.00	3.01	0.28	0.07	0.00	3.01
EST_3	0.25	0.08	0.00	2.88	0.25	0.08	0.00	2.88
EST_4	0.21	0.08	0.01	2.73	0.21	0.08	0.01	2.73
EST_5	0.42	0.09	0.00	2.87	0.42	0.09	0.00	2.87
EST_6	0.59	0.08	0.00	3.34	0.59	0.08	0.00	3.34
EST_7	0.79	0.07	0.00	3.60	0.79	0.07	0.00	3.60
EST_8	1.00	0.00	999.00	3.85	1.00	0.00	999.00	3.85
EST_9	0.85	0.07	0.00	3.85	0.85	0.07	0.00	3.85
EST_10	0.75	0.07	0.00	3.94	0.75	0.07	0.00	3.94
*ICT-Work*								
ICT1	0.87	0.05	0.00	3.50	0.87	0.05	0.00	3.50
ICT2	1.00	0.00	999.00	3.57	1.00	0.00	999.00	3.57
ICT3	0.87	0.05	0.00	3.62	0.87	0.05	0.00	3.62
ICT4	0.91	0.05	0.00	3.62	0.91	0.05	0.00	3.62
ICT5	0.83	0.05	0.00	3.74	0.83	0.05	0.00	3.74
ICT6	0.79	0.05	0.00	3.51	0.79	0.05	0.00	3.51
ICT7	0.67	0.07	0.00	3.47	0.67	0.07	0.00	3.47
ICT8	0.75	0.07	0.00	3.39	0.75	0.07	0.00	3.39
**Structural coefficients are the same across gender groups**								
ICT-Work → SRL	0.30	0.00	999.00		0.30	0.00	999.00	
ICT-Work → Self-Esteem	0.45	0.00	999.00		0.45	0.00	999.00	
SRL → Self-Esteem	0.20	0.00	999.00		0.20	0.00	999.00	
ICT-Work → ENcom	0.59	0.05	0.00		0.59	0.05	0.00	
Self-Esteem → ENcom	0.15	0.04	0.00		0.15	0.04	0.00	
SRL → ENcom	0.13	0.04	0.00		0.13	0.04	0.00	

## Discussion

This study investigated a causal relationship for understanding the role of ICT-Work in ENcom among engineering students in Thailand. In addition to the direct relationship, this relationship is also linked to two distinct factors, self-esteem and SRL. This study highlights how ICT-Work affects ENcom and tests to study whether a causal model differs between male and female students.

From a substantive perspective, the findings showed a positive relationship between ICT-Work, SRL, self-esteem, and ENcom. This finding suggested that the development of ICT-Work can directly affect Encom, help students find better learning strategies, monitor their performance, increase self-worth or think positively about themselves, and then reflect on their ENcom. Therefore, ICT-Work and ICT competence have the potential to significantly increase students’ competencies. The results are consistent with previous studies (e.g., Youssef and Dahmani [44]; Hu, Gong, Lai and Leung [39]), which pointed out that the importance of ICT-Work can improve academic performance. Previous studies have shown a positive relationship between ICT skills and academic competence in different contexts. For example, in higher education institutes, Mehrvarz, et al. [121] pointed out that digital competence could affect academic performance. Yazon, et al. [122] found that faculty’s digital literacy had a strong relationship with research output. In addition, Mangiri, et al. [123] emphasized the importance of teachers’ digital competency’s positive influence on their professionalism at vocational high school.

In view of the indirect effect of ICT-Work on ENcom, this relation links to the study of Shopova [45] and Makri-Botsari, Paraskeva, Koumbias, Dendaki and Panaikas [43]. They found that students’ abilities related to ICT skills had an impact on the learning process and professional competencies. The findings were also consistent with other studies, showing that a higher level of ICT-Work was associated with a higher level of employability potential [38, 41]. However, the findings contrasted with Wu [46], which said ICT-Work was negatively associated with learning performance.

Regarding the results of the mediating role of self-esteem and SRL, in accordance with H7, students with high ICT-Work potential have higher self-esteem, which is shown to result in increased ENcom. At the same time, having good ICT-Work competencies help students to have SRL and increases ENcom because of the student’s self-esteem. This finding is in line with SDT [30, 33], which focuses on intrinsic resources for personality development and behavioral self-regulation that are related to student performance [30, 33, 124].

Finally, the robustness of the research framework is illustrated by the results of the multigroup analyses, which indicated no gender differences in the factor loadings and structural path coefficients of ICT-Work on ENcom through self-esteem and SRL. These findings indicated a common understanding of the relationship between ICT-Work and ENcom between male and female students. The path coefficients in the male-female homologous structural model are consistent with Motahhari-Nejad [92], which indicated no gender differences in professional competencies in engineering. This shows that this proposed model can be applied well to different groups. This could imply that students of both genders recognize the necessity to develop competence in ICT-Work and ENcom as important to their engineering career life [125].

In summary, this study suggests that the positive direct and indirect effects of ICT-Work on ENcom may require time for continued development from the start of the first year of study, by beginning with fostering SRL and building self-esteem for the development of sustainable ENcom. Therefore, educational institutions or those involved in policymaking must plan and design learning activities to provide students with ICT-Work, such as being able to interact with cutting-edge software interface programs; skills in utilizing advanced computer and information technology to produce, design, and develop engineering work; or knowledge and competence in using necessary and modern information technology media variously for targeted communication.

## Limitations and directions for future research

There are some limitations of this study. First, this study used self-reporting instruments to measure ICT-Work and ENcom; thus, the results could have been influenced by self-assessment bias. Assessments by faculties or internship student supervisors may have increased the credibility of the findings. Second, the participants in this study were still studying as engineering majors at the university. Information about students’ ICT-Work provides valuable insights into the development of ENcom. However, these findings may differ from real work environments. Therefore, further research is encouraged to investigate the comparisons between the perception of students toward those factors themselves and employers on graduates’ performance in the workplace. Additional items to measure ICT-Work in future research, such as “design, install, and maintain ICT systems to support working in the professional field”, could be included in the scale. Third, a longitudinal study design would have been more suitable to capture the temporal evolution of ICT-Work development. Fourth, there were only three causal variables in this study; however, with the rapid development of ICT and given the current conditions, online learning and the use of social media are very important to the development of ICT work and ENcom. Therefore, further studies may be able to add these variables to further deepen the findings on achievement and develop ENcom. Finally, although the sample size in the present study was considered to be appropriate based on a power analysis, the findings would have been more generalizable if we had employed a larger, more representative sample from different parts of the country.

## Conclusions

This study attempted to explore the relationship between engineering students’ ICT-Work, SRL, self-esteem, and ENcom in Thailand. The results indicated that ICT-Work has a positive direct and indirect relation with self-esteem, SRL, and ENcom. Meanwhile, the mediators of the association between ICT-Work and ENcom were SRL and self-esteem. Furthermore, multigroup SEM revealed no statistically significant difference between male and female students.

The findings suggest that ICT-Work may help engineering students enhance their learning habits and self-esteem and improve their professional competencies. Educational institutions should emphasize the importance of developing engineering students in ICT-Work and the use of advanced ICT involved in the job. The results of this study may assist policymakers, educators, students, and other stakeholders in developing student ICT-Work competencies related to ENcom, self-esteem, and SRL. As ICT-Work and ICT literacy become more important for social participating and for work success, educational institutions play a crucial role in the process of integrating ICT into learning and fostering ICT-related skills among students [126].

## Supporting information

S1 Questionnaire The English and Thai version of engineering competencies, ICT competencies related to work, self-esteem, and self-regulated learning. (DOCX)

S1 Dataset (XLSX)
