# Supplementary material for: Relationships between ICT competencies related to work, self-esteem, and self-regulated learning with engineering competencies
Source: PLoS One. 2021 Dec 2;16(12):e0260659. doi: 10.1371/journal.pone.0260659 (PMC8639090; doi:10.1371/journal.pone.0260659)
Supplement: S1 Questionnaire — (DOCX) [file pone.0260659.s001.docx]

**S1 Questionnaires.**

**The English and Thai version of Engineering Competencies, ICT Competencies Related to Work, Self-Esteem, and Self-Regulated learning.**

**1) Engineering Competencies (ENcom).**

Source: Author

| **Item** | **English Version** | **Thai Version** |
| --- | --- | --- |
| ENcom1 | Can create new ideas leading to the innovation and quality of work in the profession. | สามารถสร้างสรรค์แนวคิดใหม่ เพื่อนำไปสู่การสร้างนวัตกรรมและคุณภาพของงานในวิชาชีพ |
| ENcom2 | Understand current issues and current problems in engineering in national, regional, and global levels | มีความรู้ความเข้าใจเกี่ยวกับปัญหาร่วมสมัยและความเป็นไปในปัจจุบันด้านวิศวกรรม ทั้งระดับประเทศ ภูมิภาคและโลก |
| ENcom3 | Have knowledge and skills in numerical calculation, the use of mathematical, scientific and statistical techniques sufficiently for proper use and management of engineering information correctly. | มีความรู้และทักษะการคำนวณเชิงตัวเลข การใช้เทคนิคทางคณิตศาสตร์ วิทยาศาสตร์ และสถิติเพียงพอต่อการใช้งานและจัดการข้อมูลทางวิศวกรรมได้อย่างถูกต้อง |
| ENcom4 | Can apply knowledge, theory and principles in engineering to solve the problems and make decisions in the engineering profession properly. | สามารถประยุกต์ความรู้ ทฤษฎี และหลักการทางวิศวกรรมศาสตร์ เพื่อแก้ปัญหาและตัดสินใจกับระบบงานในวิชาชีพวิศวกรรมอย่างถูกต้อง |
| ENcom5 | Can apply material science knowledge to solve engineering problems appropriately. | สามารถประยุกต์ความรู้ด้านวัสดุศาสตร์เพื่อแก้ปัญหาทางวิศวกรรมได้อย่างเหมาะสม |
| ENcom6 | Can apply engineering process, technics, and design to solve the engineering problem effectively. | สามารถประยุกต์กระบวนการ เทคนิค และการออกแบบทางวิศวกรรม เพื่อแก้ปัญหาทางวิศวกรรมได้อย่างมีประสิทธิภาพ |
| ENcom7 | Have knowledge and understanding in the impact of engineering solutions in the context of the world economy, environment and society. | มีความรู้ความเข้าใจถึงผลกระทบของการแก้ปัญหาทางวิศวกรรมในบริบทของโลก เศรษฐกิจ สิ่งแวดล้อม และสังคม |
| ENcom8 | Have perseverance and patience to complete the engineering work | มีความเพียรพยายามและความอดทน ในการทำงานด้านวิศวกรรมให้เสร็จสมบูรณ์ |
| ENcom9 | Can design and conduct experiments to solve engineering problems as well as analyzing and interpreting the meaning of data to get the correct conclusion. | สามารถออกแบบและดำเนินการทดลองเพื่อแก้ปัญหาทางวิศวกรรม ตลอดจนการวิเคราะห์และแปลความหมายข้อมูลเพื่อให้ได้ผลสรุปที่ถูกต้อง |
| ENcom10 | Can solve the problems and handle the conflicts when having to face the new situations efficiently | สามารถแก้ปัญหา จัดการความขัดแย้ง เมื่อต้องเผชิญกับสถานการณ์ใหม่ ๆ ได้อย่างมีประสิทธิภาพ |
| ENcom11 | Be aware of the quality of the work required to meet the professional standards. | ตระหนักถึงคุณภาพของงานที่ต้องได้มาตรฐานตามข้อกำหนดของวิชาชีพ |
| ENcom12 | Responsible and adhering to professional ethics to maintain the strong working ethics throughout the duration of the job as an engineer. | มีความรับผิดชอบและยึดมั่นจรรยาบรรณวิชาชีพ คงไว้ซึ่งจรรยาบรรณในการทำงานอย่างเข้มแข็งตลอดระยะเวลาการปฏิบัติงานในฐานะวิศวกร |
| ENcom13 | Understand their roles and duties and be determined to develop yourself as a professional engineer according to the professional standard of engineering and control engineering. | มีความเข้าใจบทบาทหน้าที่ของตนเอง และมุ่งมั่นที่จะพัฒนาตนเองให้เป็นวิศวกรมืออาชีพ ตามมาตรฐานวิชาชีพวิศวกรรมและวิศวกรรมควบคุม |

**2) ICT competencies related to work (ICT-Work)**

Source: Author

| **Item** | **English Version** | **Thai Version** |
| --- | --- | --- |
| ICT1 | Can interact with cutting-edge software interfaces such as human-machine interfaces, human-robot interaction, etc. | สามารถโต้ตอบกับโปรแกรมส่วนต่อประสาน (software interface) ที่ทันสมัย เช่น โปรแกรมส่วนต่อประสานระหว่างมนุษย์กับเครื่องจักร (human-machine interface) การติดต่อปฏิสัมพันธ์ระหว่างมนุษย์กับหุ่นยนต์ (human-robot interaction) เป็นต้น |
| ICT2 | Have the skills in applying digital technology (such as computers, PDAs, media players, GPS, etc.) to communicate and create professional engineering network properly. | มีทักษะในการประยุกต์เทคโนโลยีดิจิทัล (เช่น คอมพิวเตอร์, PDAs, media players, GPS, etc.) เพื่อการสื่อสารและการสร้างเครือข่ายทางวิชาชีพวิศวกรรมได้อย่างเหมาะสม |
| ICT3 | Have skills in using the advanced computer and information technology to produce, design and develop engineering work. | มีทักษะการใช้ประโยชน์จากคอมพิวเตอร์และเทคโนโลยีสารสนเทศที่ทันสมัย เพื่อการผลิต ออกแบบ และพัฒนางานวิศวกรรม |
| ICT4 | Have the skills, knowledge, and competence in using modern techniques and tools in ICT for engineering practice | มีทักษะ ความรู้ ความสามารถในการใช้เทคนิคและเครื่องมือที่ทันสมัยและเหมาะสมด้านเทคโนโลยีสารสนเทศ ในการปฏิบัติงานวิศวกรรม |
| ICT5 | Can design the working system, components, or engineering process according to the needs and requirements of the job. | สามารถออกแบบระบบการทำงาน ส่วนประกอบ หรือกระบวนการทางวิศวกรรมได้ตามความต้องการและตามข้อกำหนดของงาน |
| ICT6 | Have knowledge and competence in using necessary and modern information technology media variously for targeted communication such as project / report presentation, opinion expression, and motivation creation | มีความรู้ความสามารถในการใช้สื่อเทคโนโลยีสารสนเทศที่จำเป็นและทันสมัยได้หลากหลาย เพื่อการสื่อสารอย่างมีเป้าหมาย อาทิ การเสนอโครงการ/รายงาน การแสดงความคิดเห็น และการสร้างแรงจูงใจ |
| ICT7 | Have enthusiasm and desire to research and learn in advanced ICT to move forward to be the engineering professional that I specialize in. | มีความกระตือรือร้นและปรารถนาที่จะค้นคว้าและเรียนรู้ ด้านเทคโนโลยีสารสนเทศขั้นสูง เพื่อก้าวไปสู่ความเป็นมืออาชีพด้านวิศวกรรมที่ฉันเชี่ยวชาญ |
| ICT8 | Can further the knowledge to enhance your skills and knowledge in ICT to create more opportunities to be more professional | สามารถต่อยอดองค์ความรู้ เพิ่มพูนทักษะและความรู้ด้านเทคโนโลยีสารสนเทศของตนเอง เพื่อสร้างโอกาสที่จะเชี่ยวชาญมากขึ้น |

**3) Self-esteem**

Source: Adopted from Rosenberg, M. (1965). Society and the adolescent self-image. Princeton, NJ: Princeton University Press.

| **Item** | **English Version** | **Thai Version** |
| --- | --- | --- |
| EST_1 | On the whole, I am satisfied with myself. | โดยภาพรวม ฉันมีความพอใจในตัวฉันเอง |
| EST_2 | At times I think I am no good at all. (reverse) | บางครั้ง ฉันคิดว่า ฉันไม่มีอะไรดีเลย (reverse) |
| EST_3 | I feel that I have a number of good qualities. | ฉันรู้สึกว่าฉันเป็นคนมีความสามารถหลายอย่าง |
| EST_4 | I am able to do things as well as most other people. | ฉันรู้สึกว่าฉันเป็นคนมีความสามารถหลายอย่าง |
| EST_5 | I feel I do not have much to be proud of. (reverse) | ฉันรู้สึกว่าฉันไม่มีอะไรมากพอที่จะภูมิใจ (reverse) |
| EST_6 | I certainly feel useless at times. (reverse) | ฉันรู้สีกแน่ใจว่า ในบางครั้งฉันไม่มีประโยชน์ |
| EST_7 | I feel that I'm a person of worth, at least on an equal plane with others. | ฉันรู้สึกว่า ฉันมีเป็นคนมีคุณค่า อย่างน้อยในระดับเท่าเทียมกับคนอื่น |
| EST_8 | I wish I could have more respect for myself. (reverse) | ฉันหวังว่า ฉันจะมีความนับถือตนเองมากขึ้น (reverse) |
| EST_9 | All in all, I am inclined to feel that I am a failure. (reverse) | โดยรวมฉันมีแนวโน้มที่จะคิดว่า ตัวฉันเองเป็นคนล้มเหลว (reverse) |
| EST_10 | I take a positive attitude toward myself. | ฉันมีทัศนคติที่ดีต่อตัวเอง |

**4) Self-regulated learning (SRL)**.

Source: Adapted from Pintrich, Smith, Garcia, and McKeachie (1991)

| **Items** | **English Version** | **Thai Version** |
| --- | --- | --- |
| SRL_1 | I often feel so lazy or bored when I study for this class that I quit before I finish what I planned to do. (reverse) | บ่อยครั้งที่ฉันรู้สึกเกียจคร้านหรือเบื่อหน่าย เมื่อเรียนวิชาในสาขา และมักจะยกเลิกหรือหยุดสิ่งที่วางแผนไว้กลางคัน (reverse) |
| SRL_2 | I work hard to do well in this class even if I don't like what we are doing. | ฉันทำงานอย่างหนักในการเรียนสาขานี้ เพื่อให้ผลออกมาดีที่สุด |
| SRL_3 | When course work is difficult, I give up or only study the easy parts. (reverse) | ถ้าการบ้านหรืองานที่ได้รับมอบหมายในสาขาวิชายาก ฉันจะไม่ทำหรือเลือกทำแต่สิ่งที่ง่าย (reverse) |
| SRL_4 | Even when course materials are dull and uninteresting, I manage to keep working until I finish. | แม้ว่าเอกสารประกอบการเรียนจะไม่น่าสนุกและไม่น่าสนใจ ฉันก็ยังอ่านหรือศึกษาต่อไปจนจบ |
| SRL_5 | When studying for this course, I often look at the "clock". | ฉันมักจะดู “นาฬิกา” เมื่อฉันเข้าเรียนในสาขาวิชานี้ |
